# Magic year for multiple myeloma therapeutics: Key takeaways from the ASH 2015 annual meeting

**DOI:** 10.18632/oncotarget.13314

**Published:** 2016-11-11

**Authors:** Kejie Zhang, Aakash Desai, Dongfeng Zeng, Tiejun Gong, Peihua Lu, Michael Wang

**Affiliations:** ^1^ Department of Hematology, Zhongshan Hospital, Xiamen University, Fujian Medical University Clinic Teaching Hospital, Xiamen, China; ^2^ Department of Lymphoma/Myeloma, The University of Texas MD Anderson Cancer Center, Houston, Texas, USA; ^3^ University of Texas Health Science Center, Houston, Texas, USA; ^4^ Department of Hematology, Xinqiao hospital, Third Military Medical University, Chongqing, China; ^5^ Institute of Hematology and Oncology, Harbin first Hospital, Harbin, China; ^6^ Department of Hematology, Hebei Yanda Ludaopei Hospital, Beijing, China; ^7^ Department of Lymphoma/Myeloma, The University of Texas MD Anderson Cancer Center, Houston, Texas, USA

**Keywords:** multiple myeloma, Ixazomib, elotuzumab, daratumumab

## Abstract

Despite the availability of various anticancer agents, Multiple Myeloma (MM) remains incurable in most cases, along with high relapse rate in the patients treated with these agents. The year 2015 saw major advancements in our battle against multiple myeloma. In 2015, the U.S. Food and Drug Administration (FDA) approved three new therapies for multiple myeloma, namely Ixazomib (an oral proteasome inhibitor), Daratumumab and Elotuzumab (monoclonal antibodies against CD38 and SLAMF7 respectively). The purpose of this review is to provide a detailed analysis of these aforementioned breakthrough therapies and two other newer agents, Filanesib (kinesis spindle inhibitor) and selinexor (SINE inhibitor), presented at the 2015 annual meeting of American Society of Hematology (ASH). We also describe the role of agents targeting PD-1 axis and chimeric antigen receptor T (CAR-T) cells in the treatment of MM.

## INTRODUCTION

The neoplastic proliferation of plasma cells that produce a monoclonal immunoglobulin is a characteristic of Multiple Myeloma (MM). MM is the 2^nd^ most common hematological malignancy after lymphomas and forms 10% of all hematological malignancies in the United States [1]. The American Cancer Society predicts that about 30,330 new MM cases and 12,650 deaths caused by MM are expected in the year 2016 [2]. Despite the improvement in 5 year survival rates for patients with MM from 25% between 1975 to 1977 to 43% between 2002 and 2008 [3], this disease still remains incurable using currently available therapies. With detailed understanding of the key signaling and regulatory pathways of the tumor and the tumor microenvironment, newer agents are being discovered and evaluated in clinical trials.

The year 2015 saw major advancements in our battle against MM. In 2015, the Food and Drug Administration (FDA) approved three new therapies for MM: an oral proteasome inhibitor, ixazomib; along with two monoclonal antibodies against CD38 and SLAMF7, daratumumab and elotuzumab, respectively. In this review, we provide a detailed analysis of these breakthrough therapies and presentations at the 2015 American Society of Hematology (ASH) meeting that will ultimately alter clinical practice in MM. We describe a newer generation reversible proteasome inhibitor (ixazomib), monoclonal antibody that recognizes SLAMF7 (elotuzumab) and CD38 (daratumumab), agents targeting the PD-1 axis and chimeric antigen receptor T (CAR-T) cells along with kinesis spindle inhibitor (Filanesib) and SINE inhibitor (selinexor).

## NEW GENERATION ORAL REVERSIBLE PROTEASOME INHIBITOR

With the advent of proteasome inhibitors (PIs), the survival of an average MM patient was prolonged [4]. Bortezomib, the first PI initially approved for use in relapsed/refractory MM patients, was later extended as a first-in-line treatment of newly diagnosed MM patients [5]; however, numerous limitations complicate bortezomib treatment. Firstly, bortezomib can cause peripheral neuropathy (PN) in 37-44% of the MM patients. Also, its effectiveness is not universal as some patients are unresponsive, and the responders eventually relapse [6].

Carfilzomib is a second generation proteasome inhibitor (PI) that offers clinical advantage to several patients who relapse on bortezomib treatment. Also, this drug has a decreased incidence of PN [7]. However, drug resistance has been observed with carfilzomib treatment [7]. An important limitation to the use of both bortezomib and carfilzomib is that they have to be administered intravenously (iv), necessitating the administration of these drugs in a clinical setting, which may hamper patient’s quality of life and increases the cost of treatment [8]. However, bortezomib is now available in subcutaneous forms, which causes less peripheral neuropathic side effects.

Ixazomib (Ninlaro®), a newer generation reversible PI has clinical advantages compared with other PIs. Ixazomib acts on the 26S proteasome and inhibits the 20S catalytic subunit. At low concentrations, it reversibly inhibits the chymotrypsin-like activity of beta 5 subunit of the 20S proteasome. At high concentrations, it inhibits the caspase-like activity of beta 1 subunit and trypsin-like activity of beta 2 subunit [9] (Table 1).

**Table 1:** Review and comparison of ixazomib, bortezomib, and carfilzomib

Genetic (brand) name	Chemical structure	Mechanism of proteasome inhibition	Proteasome dissociation half-life (t_1/2_)	Administration	Treatment indication	Incidence of severe PN	US FDA approval
Bortezomib (Velcade)	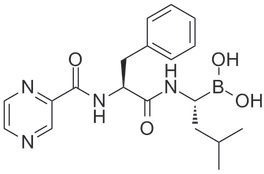	Inhibits (β1) caspase-like and (β2)trypsin-like sites of 20S proteasome, but preferentially inhibits (β5) chymotrypsin-like site	Slowly reversible β5 subunit: 110 minutes	iv/sc	First line or relapsed/refractory	High	2003
Ixazomib (Ninlaro)	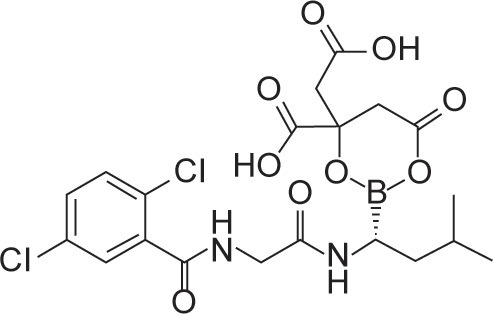	Inhibits (β1) caspase-like and (β2) trypsin-like sites of 20S proteasome, but preferentially inhibits (β5) chymotrypsin-like site	Reversible β5 subunit: 18 minutes	Oral	Relapsed/refractory	Low	2015
Carfilzomib (Kyprolis)	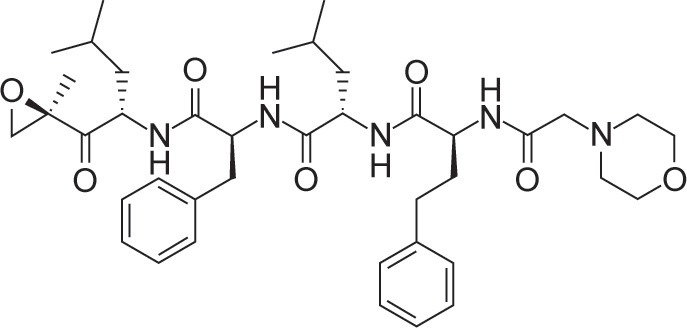	Inhibits (β1) caspase-like and (β2) trypsin-like sites of 20S proteasome, but preferentially inhibits (β5) chymotrypsin-like site	Irreversible	iv	Relapsed/refractory	Moderate	2012

Abbreviations: PI, proteasome inhibitor; iv, intravenous; sc, subcutaneous; PN, peripheral neuropathy; FDA, Food and Drug Administration

While both bortezomib and carfilzomib are given as injectable preparations, ixazomib is a novel orally administered PI approved by FDA in 2015 for use along with dexamethasone and lenalidomide for patients who were treated previously with at least one therapy. Its approval was based on the results of a randomized phase 3 TOURMALINE-MM1 study comparing treatment with ixazomib in combination with lenalidomide and dexamethasone (IRd) to treatment with lenalidomide and dexamethasone (Rd) alone. The results of which were presented at the 2015 ASH meeting [10]. Participants in the trial received one to three prior treatments. Patients refractory to previous PI- or lenalidomide-based treatment were excluded from the study. The IRd arm showed a greater progression free survival (PFS) compared to the Rd arm (20.6 months versus 14.7 months, p=0.012). The median overall survival (OS) was not reached in either arm. The median PFS in patients with unfavorable del (17) was similar to all other IRd-treated patients, suggesting that ixazomib may have a favorable effect in patients with genetic mutations. The reported side effects included rash, thrombocytopenia, leukopenia, nausea, diarrhea and fatigue, along with a few cases of PN. Given the favorable toxicity profile [11] and good manageability, the results of this orally administered chemo-free regimen are highly encouraging. The drug is being tested further as a frontline therapy, either with cyclophosphamide and dexamethasone (ICD-300 or ICD-400) or with lenalidomide and dexamethasone (TOURMALINE-MM2 study). The ORR in the study with ICD-300 and ICD-400 were 80 and 73%, respectively [12]. Currently, Ixazomib is only approved for use along with lenalidomide and dexamethasone. However, ixazomib is under testing for use as a single agent maintenance therapy in patients after autologous stem cell transplant (ASCT) and in those who have not received ASCT [9]. Thrombocytopenia was reported for ixazomib in 31% of the patients undergoing treatment [10]. Non hematologic adverse events associated with ixazomib include gastrointestinal events and rash [13].

## MONOCLONAL ANTIBODIES – A NEW ERA IN THE TREATMENT OF MULTIPLE MYELOMA

There has been a significant improvement in the survival rate of MM patients in the last 10 years [14]. Despite this progress, the median OS of patients refractory to both immunomodulatory drugs (IMiDs) and PIs is only 9 months [15, 16]. Thus, an urgent need exists for newer agents with novel mechanisms of action [17, 18]. Monoclonal antibodies (mAbs) are novel agents that have shown promising results in recent MM clinical trials [19], [20, 21]. In the current review, we will specifically focus on the monoclonal antibodies: daratumumab and elotuzumab.

### Daratumumab: Anti-CD38 monoclonal antibody

CD38 (Cluster of differentiation 38) is responsible for cellular adhesion, signal transduction and calcium signaling. CD38 expression on plasma cells is higher as compared to other hematological and solid tissues. Plasma cell tumors such as MM highly express CD38, which led to the development of therapeutic anti- CD38 antibodies [22].

**Figure 1:**
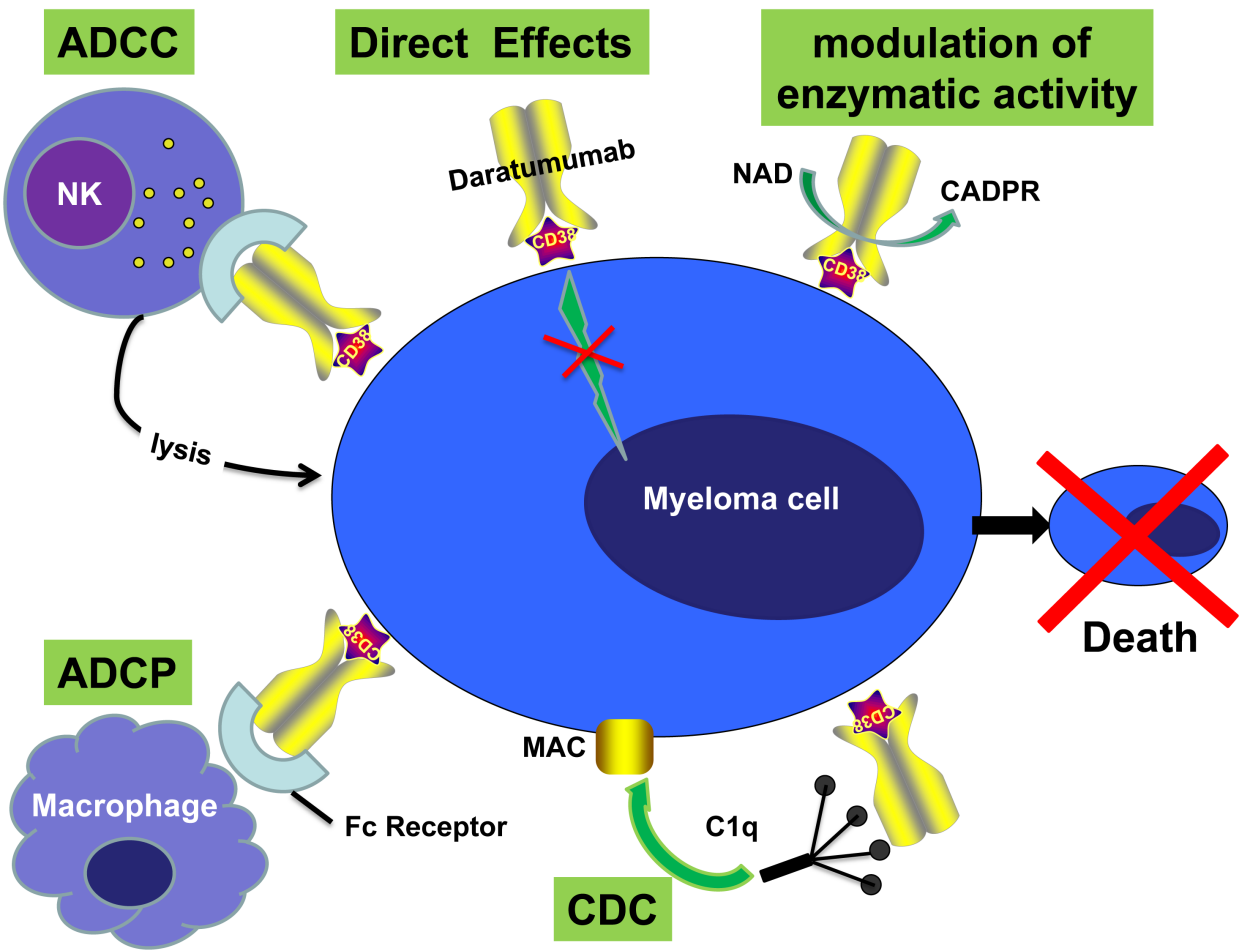
Mechanisms of action of monoclonal antibody (Daratumumab) targeting surface CD38 antigen on MM cells Daratumumab against CD38 antigen can induce tumor cell killing via Fc-dependent effector mechanisms including CDC, ADCC, and ADCP. The process of ADCC is achieved through activation of Fc receptors on myeloid and NK effector cells by tumor cell-attached immunoglobins. Subsequent cytotoxicity is mediated through ≥2 different mechanisms; one involving the release of perforin and granzymes from effector cells and the other involving death ligands FasL and tumor necrosis factor–related apoptosis-inducing ligand. In ADCP, phagocytosis of tumor cells is mediated by macrophages. CDC is dependent on the interaction of the antibody Fc domains with the classic complement-activating protein C1q leading to activation of downstream complement proteins, which results in the assembly of the membrane attack complex (MAC), that punches holes in the tumor cells. An additional result of this cascade is the production of chemotactic complement molecules C3a and C5a, which recruit and activate immune effector cells. There is also evidence that uptake of antibody-opsonized tumor cells and cellular fragments by antigen-presenting cells is associated with enhanced antigen presentation leading to tumor-specific T-cell responses. Daratumumab may also have direct effects via modulation of the activity of the targeted antigen and modulation of enzymatic activity.

Daratumumab (Darzalex), an IgG1 kappa human monoclonal antibody directed against CD38 antigen, is the first monoclonal antibody approved for use in MM. Antibody-dependent cellular cytotoxicity (ADCC), complement-dependent cytotoxicity (CDC), antibody-dependent cellular phagocytosis (ADCP), apoptosis induction via FcR-mediated crosslinking or caspase-dependent MM cell death, modulation of enzymatic activity, and immunomodulatory activity are the various mechanisms of action for Daratumumab [23–26] (Figure 1). In a phase II trial, single agent daratumumab showed approximately 30% response rate in heavily pretreated MM patients [27]. In 2015, Daratumumab, was approved by the FDA [28] to treat patients with MM who had received at least 3 prior therapies including either a PI or an IMiD, or those who are double-refractory to these drugs.

At the 2015 ASH meeting, researchers reported results of a phase 1/2 study (GEN503) of Darzalex in combination with dexamethasone and lenalidomide in patients who had relapsed or refractory (R/R) MM [29]. After 7.8 months of median follow up, an 88% ORR was recorded. The PFS and OS were not achieved. The type and rate of infusion-related reactions were similar to those reported in studies of Darzalex monotherapy. Toxicity was common but was not increased as compared to that caused by lenalidomide/dexamethasone. The combined analysis of the GEN501 and SIRIUS trials that studied daratumumab as a monotherapy in R/R MM (relapsed or refractory multiple myeloma) created much interest [30]. Despite the ORR being 31%, a median OS of 20.1 months was reported on the combined analysis of the two trials. The median OS was not reached among the responders, while in those with minimal or no response, OS was reported to be 17.5 months, which favorably compared with historical controls. Another open label, multicenter study of Darzalex investigated the drug in combination with Pomalyst (pomalidomide, Imnovid) and dexamethasone in R/R MM patients with at least two lines of prior therapy [31]. The ORR was 58.5%, including a 57.5% response rate in patients who were double-refractory to a PI and an IMiD. After a median follow-up of 4.2 months, the six-month estimated PFS was 66%, and no additional safety concerns were noted. The most common hematological side effects of any grade (≥20%) were anemia, thrombocytopenia, and neutropenia [27, 31].

### Elotuzumab: Anti-CS1 monoclonal antibody

CS1 (cell-surface glycoprotein CD2 subset 1), also known as SLAMF7, is a cell surface glycoprotein receptor located on chromosome 1q23 [32]. SLAMF7 expression is high in myeloma cells and natural killer (NK) cells but not on normal tissues, making CS1 a potential target for MM immunotherapies [33].

**Figure 2:**
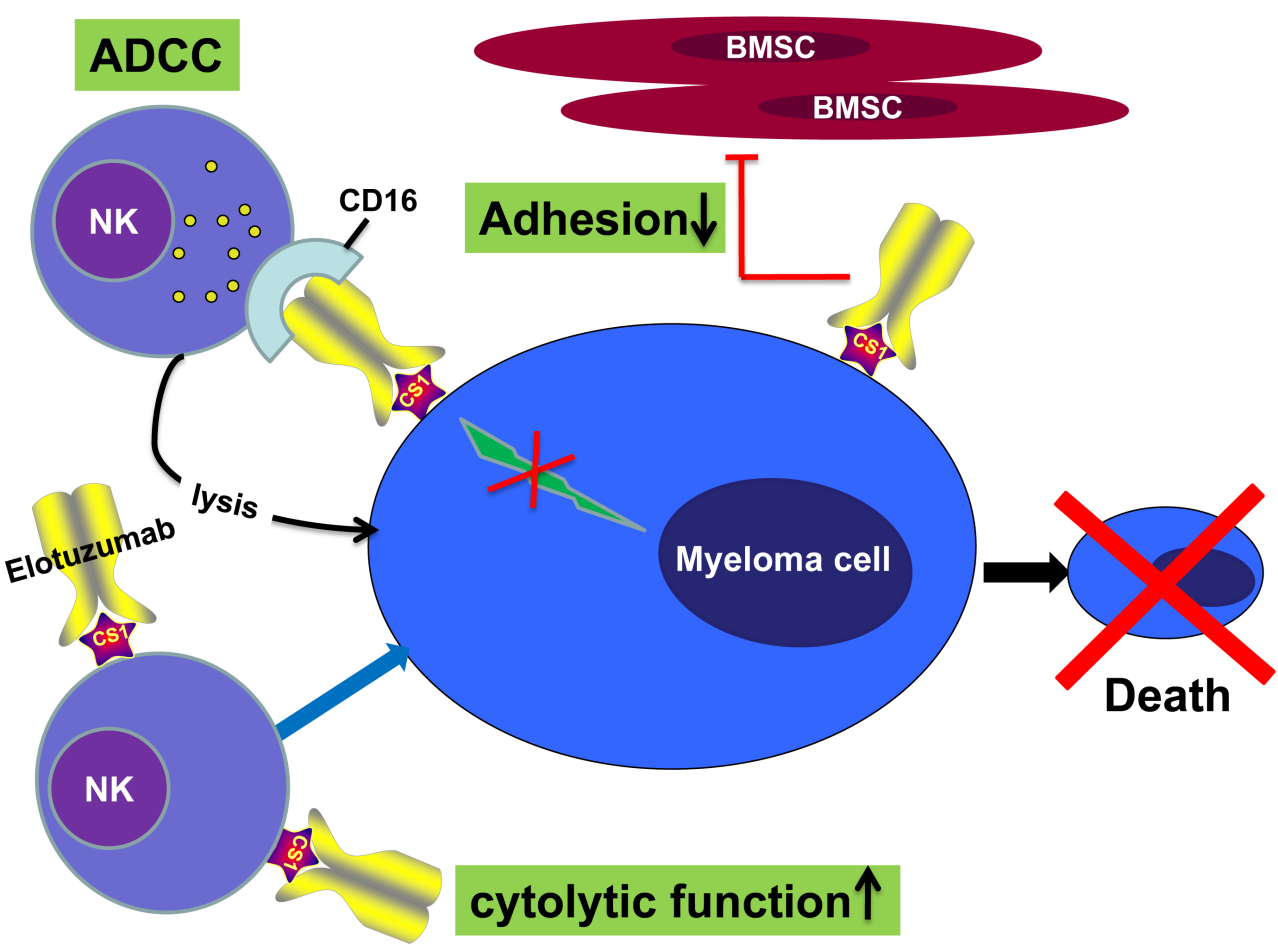
Mechanisms of action of elotuzumab The primary mechanism of action of elotuzumab against myeloma cells is NK cell-mediated ADCC. Elotuzumab can also interfere with the adhesion of myeloma cells to bone marrow stromal cells (BMSC), or can induce NK cell activation directly through binding CS1 expressed on NK cells.

Elotuzumab (Empliciti™) is a humanized immunoglobulin (Ig) G1-k monoclonal antibody targeting CS1. NK cell-mediated ADCC through the CD 16 pathway is the predominant elotuzumab mechanism of action [33]. Elotuzumab activates natural killer cells through Fc receptors and the SLAMF7 pathway. After binding to CS1, elotuzumab binds to CD16 on NK cells, activating the NK cell and leading to release of cytotoxic granules, eventually leading to MM cell death [34]. CS1 activates NK cells by coupling with EWS/FLI1 Activated Transcript 2 (EAT-2). In MM cells, CS1 signaling is compromised due to absence of EAT-2 expression; thus, it does not affect the proliferation of MM cells [35, 36]. Elotuzumab also impairs adhesion of MM cells to bone marrow stromal cells (BMSCs) [37](Figure 2).

Elotuzumab lacks single-agent activity, unlike the anti-CD38 antibodies. However, this antibody has synergistic activity when combined with dexamethasone plus lenalidomide (Rd) [38]. The combination of elotuzumab with Rd showed a 30% risk reduction for disease progression and death in patients with R/R MM. These interim results of a landmark ELOQUENT-2 phase 3 trial, presented at ASCO 2015, are the largest analysis of a monoclonal antibody in MM and the first positive findings for a targeted immunotherapy approach in a phase 3 clinical trial in patients with MM. Based on this study, elotuzumab was declared a breakthrough therapy by the U.S. FDA for patients who have received ≥1 previous therapies for MM [39].

At the ASH meeting, updated data provided longer follow-up results of the ELOQUENT-2 phase 3 trial [40]. Previously, at a median follow-up of 24 months, the PFS rates were 41% in the triple-therapy arm of ERd and 27% Rd alone (*P* = .004). At 1 year, the PFS rates were 68% and 57%, respectively. The OS rates were 79% and 66% (P = .002), respectively. In the ASH update, the 3-year PFS rates were 26% and 18% in the two arms, respectively. A time-to-next-treatment analysis favored the Empliciti arm (33 months versus 23 months). Interim OS analysis showed a trend in favor of ERd. Furthermore, a phase 2 randomized study of lenalidomide and dexamethasone combined with elotuzumab versus lenalidomide and dexamethasone without elotuzumab showed promising results as well [41].The median PFS figures were 9.9 months versus 6.8 months. The two-year follow-up showed a 24% reduction in the risk of disease progression, and OS analysis showed a 25% reduction in the risk of death, with no significant increases in adverse events. However, being a phase 2 study, the trial was not powered to assess the true benefit of elotuzumab in combination with lenalidomide and dexamethasone. Of note, elotuzumab activity against disease with high risk cytogenetic features such as t (4; 14) and del (17p) has been reported [42]. These patients typically have less benefit from conventional therapies. The common adverse events for elotuzumab are hematological adverse events. In Lonial et al’s study 34% of patients had neutropenia (grade 3/4) in elotuzumab group versus 44% in the control group; lymphocytopenia (grade 3/4) was reported in 77% and 49% of patients, respectively [42].

Up until this point, we have analyzed the three MM therapies newly approved by the U.S. FDA. The pivotal efficacy results and the main toxicities of these are shown in Table 2.

**Table 2:** Selected studies with ixazomib, elotuzumab and daratumumab in relapsed/refractory MM

Study	Type of study	Regimen	Schedule	N	Prior treatment	Response	TTE	Key toxicities
TOURMALINE-MM1 Moreau P, et al8	Phase 3	Ixazomib Revlimid dexamethasone vs Revlimid dexamethasone	ixazomib 4 mg, PO d 1, 8, 15 lenalidomide 25mg PO d 1-21 dexamethasone 40mg PO d 1, 8, 15,22, In 28 d cycles	722	RRMM after 1-3 prior lines of therapy bortezomib 69% thalidomide 45% lenalidomide 12%	IRd CR:11.7% ≥VGPR:48.1% ORR:78.3% Rd CR:6.6% ≥VGPR:39% ORR:71.5%	IRd Median PFS: 20.6 mos., OS: No results provided Rd Median PFS: 14.7 mos., OS: No results provided	IRd ≥ grade 3: neutropenia 19% anemia 9% thrombocytopenia 13% pneumonia 6% diarrhea 6% nausea 2% vomiting 1% PN 2% rash 4% renal failure 2% heart failure 2% without a substantial increase in overall toxicity than Rd
NCT02046070 Dimopoulos MA et al10	Phase 2	Ixazomib Cyclophosphamideide Dexamethasone	ixazomib 4 mg PO d 1, 8, 15 cyclophosphamide 300 mg/m2(ICd-300 arm) 400mg/m2 (ICd-400 arm) PO d 1, 8, 15 dexamethasone 40mg PO d 1, 8, 15,22, In 28 d cycles	70 (Transplant- Ineligible)	NDMM	ICd-300 CR:10% PR: 70% VGPR:17% ORR:80% SD:6% ICd-400 CR:3% PR: 19% VGPR:4% ORR:73% SD:8%	No results provided	≥ grade 3: ICd-300 53% ICd-400 62% Serious ICd-300 33% ICd-400 53% Most common≥ grade3 AEs were neutropenia, Anemia, pneumonia
ELOQUENT-2 Dimopoulos MA, et al32 Lonial S, et al34	phase 3	elotuzumab lenalidomide dexamethasone vs lenalidomide dexamethasone	Elotuzumab: iv 10 mg/kg d1, 8, 15,22 for cycles 1-2 10 mg/kg d1, 15, starting with cycles 3 lenalidomide : PO 25mg d 1-21 dexamethasone: once weekly 8 mg iv and 28 mg po, on elotuzumab days 40 mg po on other days In 28 d cycles Len: 25 mg on days 1-21 Dex: 40 mg once weekly In 28 d cycles	646	RRMM Median: 2 Range: 1-4 thalidomide: 48% lenalidomide (not refractory): 6% bortezomib: 70%	≥PR: 79% VGPR: 28% CR: 4% ≥PR: 66% VGPR: 21% CR: 7%	Median PFS: 19.4 mos. PFS at 3 years: 26% OS at 1 year: 79% Median PFS: 14.9 mos. PFS at 3 years: 18% OS at 1 year: 66%	Grade 3/4 lymphopenia: 78% neutropenia: 35% anemia: 20% thrombocytopenia: 21% Herpes zoster: 4.1 per 100 patient-years Infections (any grade) :83% IRR: 10% (mostly grade 1/2) Grade 3/4 lymphopenia: 49% neutropenia: 44% anemia: 21% thrombocytopenia: 20% Herpes zoster: 2.2 per 100 patient-years Infections (any grade) :75%
NCT01478048 Palumbo A, et al33	phase 2	elotuzumab bortezomib dexamethasone vs bortezomib dexamethasone	Elotuzumab: iv 10 mg/kg d1, 8, 15,22 for cycles 1-2 10 mg/kg d1, 11 for cycles 3-8 10 mg/kg d1, 15 starting with cycles 9 bortezomib : iv/ih 1.3 mg/m2 d1, 4, 8,11 for cycles 1-8 1.3 mg/m2 d1, 8,15 starting with cycles 9 dexamethasone: 8 mg iv and 8 mg po, on elotuzumab days 20 mg po on other days In 21-day cycles for cycles 1-8 and then in 28-day cycles bortezomib : iv/ih 1.3 mg/m2 d1, 4, 8,11 for cycles 1-8 1.3 mg/m2 d1, 8,15 starting with cycles 9 dexamethasone: 20 mg po In 21-day cycles for cycles 1-8 and then in 28-day cycles	152	RRMM Median: 1 Range: 1-3 Prior IMiD: 74% Prior PI (not refractory): 52	CR:4% VGPR:33% PR: 30% ORR:66% MR:5% SD:17% PD:5% NE:7% CR:3% VGPR:23% PR: 36% ORR:63% MR:7% SD:19% PD:5% NE:7%	Median PFS: 9.7 mos. OS at 1 year: 85% OS at 2 year: 73% Median PFS: 6.9 mos. OS at 1 year: 74% OS at 2 year: 66%	Any grade (≥ grade 3) All 100% (71%) Infection 67% (21%) Diarrhea 44% (8%) Constipation 40% (1%) Cough 44% (1%) Anemia 37% (7%) Peripheral neuropathy 36% (9%) Pyrexia 37% (0%) peripheral edema 29% (4%) Insomnia 29% (1%) Asthenia 28% (4%) Fatigue 29% (4%) Paresthesia 27% (0%) Nausea 27% (1%) thrombocytopenia 16% (9%) IRR in elotuzumab group: 7% (all grade 1/2); no additional events in elotuzumab group compared with control group All 96% (60%) Infection 53% (13%) Diarrhea 33% (4%) Constipation 29% (0%) Cough 24% (0%) Anemia 29% (7%) Peripheral neuropathy 36% (12%) Pyrexia 28% (4%) peripheral edema 24% (0%) Insomnia 19% (1%) Asthenia 29% (3%) Fatigue 25% (1%) Paresthesia 19% (5%) Nausea 21% (1%) thrombocytopenia 27% (17%)
SIRIUS Lonial S, et al22	phase 2	daratumumab	Initially daratumumab 8 or 16 mg/kg; 16 mg/kg was established as the recommended dose for further study. Daratumumab 16 mg/kg was given weekly for 8 weeks, every 2 weeks for 16 weeks, followed by monthly infusions	Daratumumab 16 mg/kg: 106	RRMM Daratumumab 16 mg/kg; Median: 5 Range: 2-14 Thal: 44% Len-refractory: 88% Bort-refractory:90% Carf-refractory:48% Pom-refractory:63%	Daratumumab 16 mg/kg; ≥PR: 29.2% VGPR: 9.4% CR: 2.8%	Daratumumab 16 mg/kg; Median PFS: 3.7 mos. OS at 1 year: 64.8%	Daratumumab 16 mg/kg; Any grade fatigue 40% anemia 33% IRR: 43%; mostly grade 1/2 (grade 3 in 5%)
GEN503 Plesner T, et al24	Phase 1/2	daratumumab lenalidomide dexamethasone	MTD not reached; highest dose-level: Daratumumab 16 mg/kg iv weekly for cycles 1- 2, every other week for cycles 3-6 monthly starting with cycle 7 Len: 25 mg po days 1-21 Dex: 40 mg weekly In 28 d cycles	Phase 2 with daratumumab 16 mg/kg: 32	RRMM Phase 2 with daratumumab 16 mg/kg; Median: 2 Range: 1-3 Thal: not reported Len (not refractory): 34% Bort: not reported	Phase 2 with Daratumumab 16 mg/kg; ≥PR: 88% VGPR: 28% CR: 25%	Phase 2 with daratumumab 16 mg/kg Not reported	Phase 2 with daratumumab 16 mg/kg; IRR: 56%, mostly grade 1/2 (grade 3 in 6%)
NCT01998971 Chari A, et al26	Phase 1b	daratumumab pomalidomide dexamethasone	Daratumumab 16 mg/kg iv weekly for cycles 1- 2, every other week for cycles 3-6 monthly starting with cycle 7 Pom: 4 mg po d1-21 Dex: 40 mg weekly In 28 d cycles	77	RRMM Median: 3.5 Range: 2-10 Thal-refractory: not reported: Len-refractory: 88% Bort-refractory:65% Carf-refractory:30% PI and IMID- refractory: 65%	53 patients with >1 post-baseline assessment; ≥ PR: 58.5% VGPR: 23% CR: 8% Double-refractory patients (n=40); ≥PR: 57.5%	No results provided	IRR: 61%; little additional toxicity when Daratumumab was added to pom-dex

RRMM, relapsed/refractory multiple myeloma; NDMM, newly diagnosed multiple myeloma; bort, bortezomib; carf, carfilzomib; dex, dexamethasone; pom, pomalidomide; len, lenalidomide; PI, proteasome inhibitor; thal, thalidomide; CR, complete response; PD, progressive disease; PR, partial response; VGPR, very good partial response. SD, stable disease; TTE, time to events; ORR, overall response rate was defined as partial response or better; NE, not estimate; PFS, progression-free survival; OS, overall survival; IRR, infusion-related reaction;

## IMMUNE CHECKPOINT INHIBITORS TARGETING PD-1/PD-L1 AXIS

**Figure 3:**
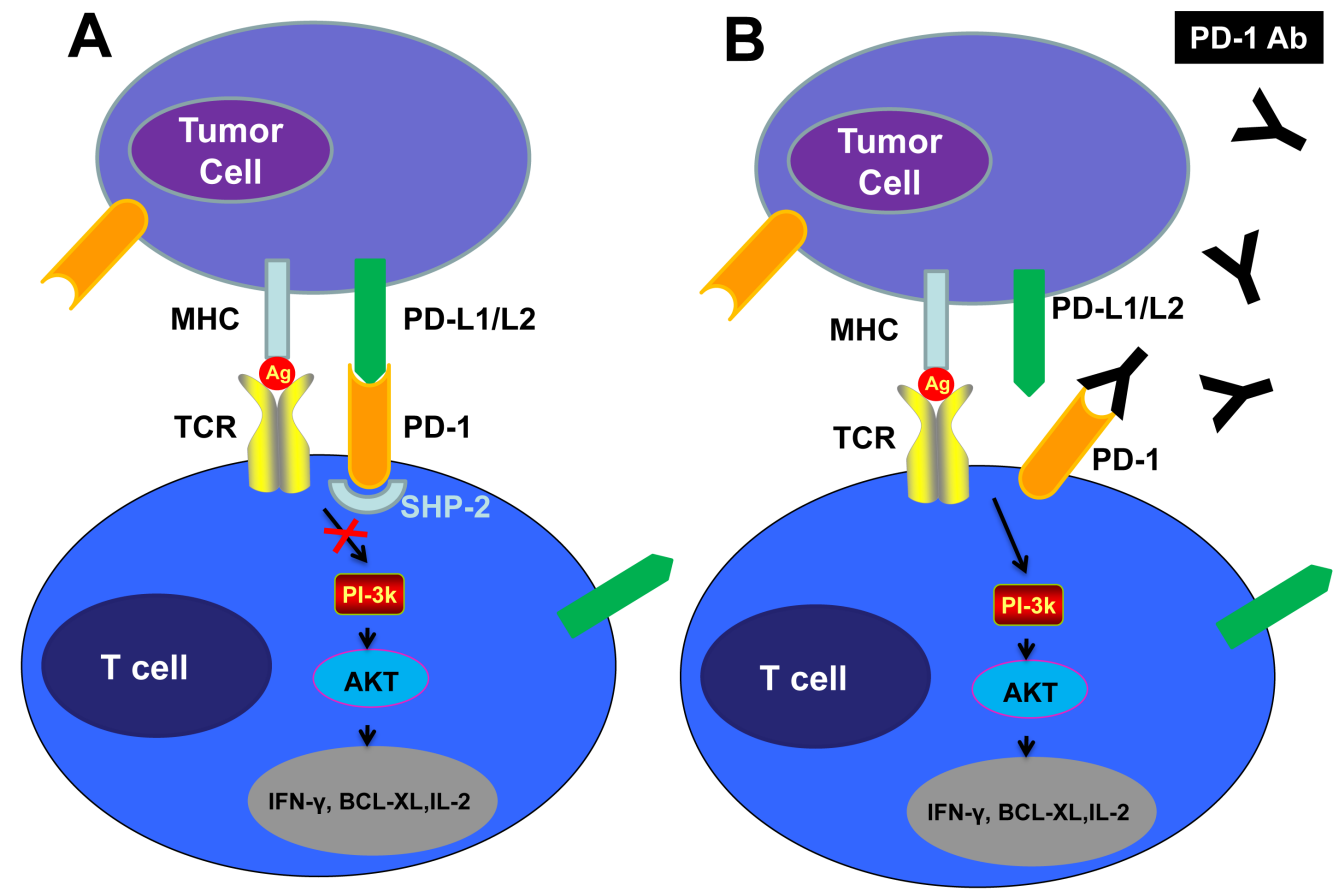
Checkpoint Inhibition via the PD-1 Pathway “Put the Brakes on” the Antitumor Response, While PD-1 or PD-1-Blocking Antibodies Release the Brakes A, PD-L1 expressed on tumor cells binds to PD-1 on T-cells recruiting a phosphatase, SHP-2, which blocks the PI3K pathway and leads to down-regulation of T-cell survival proteins, including IFN-γ, BCL-XL and IL-2, resulting in T-cell anergy or “exhaustion”, thereby “braking” the T-cell immune response. B, The anti-PD-1 antibodies block the PD-1 pathway “release the brake” preventing suppression of the anti-tumor response.

A member of the B7 receptor family, Programmed Death-1 (PD-1), has a significant role in immune regulation. The PD-1 receptor is a member of the immunoglobulin superfamily and is a 288-amino acid type I transmembrane protein [43, 44]. PD-1 is upregulated on activated macrophages, B cells, T cells, NK cells, NKT cells, and dendritic cells (DCs) [43]. Binding of PD-1 to PD-L1 (B7-H1) and PD-L2 (B7-DC) ligands causes activated T-cell apoptosis via negative signaling [45]. The higher expression of SHP-2, a cytoplasmic SH2 domain containing protein tyrosine phosphatase, causes the inhibition of the PI3K pathway and the subsequent inhibition of AKT. This results in a decreased production of Bcl-xL, a molecule associated with the intrinsic apoptotic pathway [43] (Figure 3). The end result of the PD-1 pathway is immune tolerance [46]. Cancer cells utilize the PD-1 pathway through expression of PD-L1 on tumor-infiltrating lymphocytes (TILs) [47] leading to impairment of anti-tumor responses [48]. Thus, antibodies targeting the PD-1 axis “release the brakes” on T-effectors causing anti-tumor cytotoxicity [49]. The presence of PD-1 on T-regulatory (T_reg_), B- and NK-cells enhances anti-tumor cytotoxicity through increased NK cell-mediated killing and Treg suppression via PD-1 blockade [50, 51].

Currently, the use of anti PD-1 agents is a “hot topic” in cancer therapeutics. Two widely marketed anti PD-1 agents, pembrolizumab and nivolumab (IgG4 isotype antibodies), have both been approved in squamous non–small-cell lung cancer and melanoma. PD-1 blockade as a treatment option in MM has been investigated in various clinical trials, with disappointing results in general [52]. 27 patients with R/R MM were enrolled in an active phase I trial of nivolumab in hematologic malignancies. The preliminary results of the trial were reported at the 2014 ASH Annual Meeting [53]. Although no objective response was observed in this study, 18 patients (67%) had stable disease, and the PFS at 24 weeks was 15%.

At the 2015 ASH meeting, pembrolizumab (KEYTRUDA) showed a much more robust response when combined with immune-modulatory drugs. Pembrolizumab was tested as a combination therapy with lenalidomide and dexamethasone for patients with R/R MM in the KEYNOTE-023 phase 1 trial [54]. An ORR of 76% was observed with a recommended fixed dose of 200 mg. In another study presented at the ASH meeting, Pembroluzimab was combined with pomalidomide and dexamethasone in patients with R/R MM [55]. The trial enrolled 33 patients, of whom 23 (70%) were refractory to an immunomodulatory drug and a proteasome inhibitor. An ORR of 60% in 27 evaluable patients was observed. The response rate was 55% in 20 patients who were refractory to both IMiD and PI.

## CHIMERIC ANTIGEN RECEPTOR (CAR) T- CELL THERAPY

Single CAR T-cell therapy is a massive advancement in the development of immune therapy against cancers. CAR T-cells, developed to express the antigen-binding domain from a B cell receptor that is fused to the intracellular domain of a CD3 T-cell receptor (CD3-zeta), have shown activity in CD-19 related disease [56]. Although 95% of MM patients do not express CD19 on their tumor cells, CAR T-cells have been used effectively to induce remission in MM patients. One way to improve the outcomes of CAR T-cell treatment in MM patients is to develop effective immunotherapies targeting antigens expressed by MM cells. A fitting target for CAR T-cell therapy of MM had not been previously identified because most proteins expressed on MM cells are also expressed on essential normal cells. Both B-cell maturation antigen (BCMA) and CS1 have been identified as promising immunotherapeutic targets in MM [57, 58]. BCMA is expressed in mature B cells and plasma cells and promotes the survival of long-lived plasma cells [59]. BCMA expression is high in most, if not all, MM cells. A wide range screening of normal human tissues by immunohistochemistry revealed no expression of BCMA except in lymphoid tissue, suggesting that an anti-BCMA CAR T-cell therapy will have favorable efficacy in MM [57].

At the ASH 2015 meeting, impressive results were reported by Dr. Kochenderfer regarding novel CAR T-cell therapy targeting BCMA [60]. This dose-escalating phase 1 trial enrolled a total of 12 heavily pretreated patients. Each patient received a single infusion of their modified T cells after first being administered cyclophosphamide 300 mg/m2 and fludarabine 30 mg/m2 for three days. Among the two patients who received the maximum dose, one patient showed a complete and durable remission. However, sings of cytokine release syndrome occurred, necessitating management.

## OTHER NOVEL AGENTS

### Filanesib (kinesin spindle inhibitor)

Filanesib (Arry-520) is a kinesin spindle protein inhibitor that arrests cells undergoing mitosis and promotes apoptosis [61]. In highly refractory MM patients, single agent Filanesib resulted in a 16% PR and when in combination with dexamethasone, the PR increased to 22%. Filanesib in combination with low dose dexamethasone has shown a manageable safety profile [62]. The drug is also being tried in other combinations with PIs (bortezomib or carfilzomib). A phase I study in combination with bortezomib reported an ORR of 42% and the combination was found to be well-tolerated [63]. Shah et al. reported a 42% ORR (≥ PR) and 52% clinical benefit rate (≥ MR) for the combination with carfilzomib in the phase 1 study [64] [53]. Zonder et al. reported an ORR of 30% for a combination with carfilzomib in 72 patients with at least 2 prior regimens with BTZ and IMiD. The most common hematologic toxicities included anemia, neutropenia and thrombocytopenia [65].

### Selinexor (SINE inhibitor)

Selinexor (KPT-330) is a novel member of the selective inhibitors of nuclear export (SINE) class of compounds. KPT-330 has significant activity against hematological malignancies, especially those that are refractory to standard chemotherapeutic agents [66]. In a phase I dose escalation study (NCT01607892) conducted by Chen et al., 40% showed minimal response, 6.7% partial response and 33% had stable disease among the 17 heavily pretreated relapsed or refractory MM patients. The most common side effects were anorexia, nausea, vomiting, diarrhea, thrombocytopenia and neutropenia. These side effects were well managed with supportive care [67]. A phase I study of selinexor combined with carfilzomib and dexamethasone to evaluate both efficacy and tolerance is currently underway (NCT02199665) [68, 69]. The early results show a 75% PR without any unexpected toxicities in those refractory to carfilzomib, suggesting that this regimen overcomes carfilzomib resistance [70]. Also phase 1/2 clinical trials in combination with dexamethasone, liposomal doxorubicin, pomalidomide/dexamethasone, or PIs/dexamethasone are ongoing, with promising interim results (NCT02336815, NCT02186834, or NCT02199665, respectively) [71].

In conclusion, 2015 has been an exciting year for relapsed/refractory MM, with three drugs approved by U.S. FDA - namely ixazomib, elotuzumab and daratumumab. The first two drugs were approved for use with dexamethasone and lenalidomide in patients with one prior line of therapy. Therefore, now we have three options in the patients with one to three prior lines of treatment. Data have been reported on carfilzomib being combined with lenalidomide/dexamethasone and on ixazomib and elotuzumab being combined with lenalidomide/dexamethasone. On the other hand, daratumumab was approved for use as a single agent for patients with double refractory disease or those who have had more than three lines of therapy.

It is difficult to predict which of these drugs will be a frontrunner in the future therapy of MM. The choice of optimal combination therapy can be made only after giving due consideration to various factors. These factors include (1) treatment-related factors: sensitivity and tolerability to prior treatments; (2) disease-related factors: disease presentation and cytogenetics; and (3) patient-related factors: age, organ failure and comorbidities. Head-to-head comparisons are required to explore the efficacy of these novel treatments in certain subgroups of patients. Based on the current data, we suggest that carfilzomib and ixazomib combinations may be utilized for people who require fairly rapid cytoreduction. Particular ixazomib combinations may be utilized for older patients because ixazomib is remarkably well tolerated, and elotuzumab may be an option that is suitable for slow biochemical relapses or patients who have other comorbidities because of elotuzumab’s fairly decent safety profile. Daratumumab is a good choice for heavily treated or late relapse patients. Based on daratumumab’s single-agent activity, it is also being brought early into the clinical setting.

Several trials are in progress with the goal of further improvement in clinical outcomes for our patients with MM. Thus, we believe that we are at the verge of another paradigm shift in the management of MM.
